# Dietary Protected Sodium Butyrate and/or Olive Leaf and Grape-Based By-Product Supplementation Modifies Productive Performance, Antioxidant Status and Meat Quality in Broilers

**DOI:** 10.3390/antiox12010201

**Published:** 2023-01-15

**Authors:** Almudena de-Cara, Beatriz Saldaña, Patricia Vázquez, Ana I Rey

**Affiliations:** 1 Departamento Producción Animal, Facultad de Veterinaria, Universidad Complutense de Madrid, Avda. Puerta de Hierro s/n., 28040 Madrid, Spain; 2 Nuevas Tecnologías de Gestión Alimentaria S.L., C/Marconi, 9, Coslada, 28823 Madrid, Spain; 3 Imasde Agroalimentaria S.L., C/Nápoles, 3, Pozuelo de Alarcón, 28224 Madrid, Spain

**Keywords:** antioxidant status, meat quality, performance, phytobiotics, protected sodium butyrate

## Abstract

To meet the demand for chicken meat production, new additives that promote growth and health without adverse effects on meat quality are being investigated. This study was conducted to investigate the effect of protected sodium butyrate (PSB) (0 vs. 2 g/kg), an olive leaf and grape-based by-product (OLG-mix), or a combined supplementation of PSB and OLG-mix on productive performance, antioxidant status, carcass, and meat quality in broilers. PSB improved performance parameters with greater effect in the initial phase. Both, PSB and OLG-mix increased the plasma superoxide dismutase (SOD); however, PSB supplementation was more effective to delay the lipid oxidation of meat from the initial day of storage. OLG-mix produced meat with greater color intensity, b* value and lesser drip losses than PSB. The combination of PSB + OLG-mix did not produce more marked effects that the individual administration; except to control the oxidation of meat. Linear and positive correlations between antioxidant enzymes and weight gain were observed. Significant linear and negative relationships were quantified between plasma SOD and meat lipid oxidation according to dietary treatment. Therefore, the present study would be a first approximation to the possibilities for predicting growth range and meat quality through the evaluation of the blood oxidative status.

## 1. Introduction

According to the OECD/FAO Agricultural Outlook, the global meat will reach 374 Mt by 2030 mainly because of the growth in poultry production [1]. Consumers are attracted to poultry meat due to its low price as well as its high-lean and low-fat content compared to the meat of other species [2]. To meet the high demand for chicken meat production, antibiotics in the animals’ diets at sub-therapeutic doses have been widely used for many years as growth promoters (AGPs) to maintain expected growth indexes [3,4]. Nevertheless, nowadays this use has been banned in several countries [4]. Therefore, new feeding strategies are being investigated, such as the use of substances with antioxidant activity that, due to their positive effects on animal health, could promote growth.

Among the new substances tested highlight the use of organic acids, short or medium chain fatty acids, and plant or fruit by-products. Butyric acid (BA) is a volatile short-chain fatty acid that is produced within the intestinal lumen by bacterial fermentation of different components of diet, mainly carbohydrates, and serve as energy provider for intestinal cells [5]. This compound is frequently used in animal nutrition in the form of sodium butyrate (SB) because it is solid, stable, and less odorous [6]. When SB is protected (PSB) it reaches the proximal small intestine without dissociation, and it is absorbed like a source of energy for gastrointestinal epithelial cells, increasing both crypt depth and villus height [7,8]. In the form of PSB, some authors have also found improvement in the activity of digestive enzymes, pancreatic secretions, and nutrient digestibility, which would be related to the improvement in the productive performance of broilers found in some studies [9,10,11,12,13]. Other authors have reported that PSB presents antioxidant properties in vivo through the increase in antioxidant enzymes at the cellular level [14], with effective control in oxidative stress in hostile situations or heat stress [14,15]. Moreover, some recent investigations have found that dietary supplementation in the form of PSB at 1.2 g/kg or 1 g/kg may improve lipid stability of the meat once the animal is dead [15,16]. However, studies on the effects of PSB supplementation on chicken meat characteristics, and especially, how it can modify the shelf life are limited in the literature [15,16]. Considering that poultry meat stands out for its high proportion of polyunsaturated fatty acids (PUFAs) that translate into greater oxidative deterioration [17], the effects of incorporating PSB into the diet deserve more attention. This is especially interesting because there are different forms of PSB in the feed market and the effects on the shelf life of meat could vary according to the dose or the form of presentation.

In addition, fruit by-products, herbs, or plant-based supplements are rich in bioactive secondary metabolites [18,19]. Consequently, they have been used in several industries and could be a natural alternative for replacing AGPs because of their antibacterial, anti-inflammatory, immunomodulatory, and digestive stimulatory effects [19]. Moreover, several antioxidants of plant origin have been investigated and used as effective protective agents against free radicals induced by oxidative stress [20]. Their positive effects have been extended to meat quality since plants or its derived extracts could inhibit process of lipid oxidation, development of rancidity and off-flavors, and improve color stability [21,22,23,24]. However, there is not any study that combines the possible effects of PSB and plant or fruit-based by-products on broilers.

It is hypothesized that the administration of PSB could produce similar effects than an additive based on olive leaf and grape by-products, and the combination of both (PSB and phytobiotic extract) could improve the productive performances, the oxidative status, and the quality of the meat. Therefore, the aim of this study was to investigate the effect of dietary PSB (2 g/kg feed), an olive leaf and grape-based by-product (2 g/kg) (OLG-mix), and the PSB combination with OLG-mix on growth performance in broilers from 1 to 40 days of age and carcass yield, oxidative status, drip losses, color, and stability of the breast muscle at 40 days of age.

## 2. Materials and Methods

### 2.1. Animals Ethics

All the experimental procedures performed in this study complied with Spanish policy for Animal Protection [25], which is in accordance with the European Union Directive 2010/63/UE on the protection of animals used in research [26]. The experimental procedures were reviewed and approved by the Ethics Committee on Animal Testing of University of Murcia, Spain (CEEA: 413/2018).

### 2.2. Birds, Husbandry, and Experimental Design

The study was carried out at the Imasde Agroalimentaria Broiler Center, located at Faculty of Veterinary Science (University of Murcia, Murcia, Spain). A total of 1320 one-day-old male Ross 308 broilers were weighed individually (48.29 ± 0.09 g) and allotted in groups of 22 broilers in 60 pens. Each pen was 1.83 m^2^ in size (1.58 × 1.16 m^2^) and was equipped with a pan feeder and a nipple drinker and wood shavings were used as litter. The temperature of the house was maintained at 34 °C as an average for the first week and decreased from the second week by 3 °C per week until it reached 22 °C with a relative humidity of 50–70%, following the recommendations of Ross 308 Management Handbook [27]. The building was closed, without windows, and was provided with artificial programmable light. The applied light program was 23:1 (light:dark) during the first week of life (3040 lux of fluorescent light intensity at bird eye), and then 18:6 (light:dark) during the rest of the breeding (10 lux of fluorescent light intensity at bird eye). Electric heating system and forced ventilation was used. Concentration of CO_2_ and NH_3_ did not exceed the EU standards.

The experimental design was completely randomized with four treatments in a 2 × 2 factorial arrangement with two inclusion levels of PSB (0 vs. 2 g/kg) and two inclusion levels of OLG-mix (0 vs. 2 g/kg). Each treatment was replicated 15 times and the experimental unit for analysis of all data was the pen (22 broilers per pen).

### 2.3. Diets

The feeding program consisted in three types of diets that were supplied from 0 to 12, 13 to 28, and 29 to 40 days of age. Within each feeding period, one basal diet was manufactured with wheat, soybean meal, and corn as primary ingredients (Appendix A1). The basal diet split equally into four subsamples that were supplemented with the experimental products resulting in the following dietary treatments: Control diet without any added supplement, CON; 2 g/kg of PSB; 2 g/kg of OLG-mix; and 2 g/kg of PSB + 2 g/kg of OLG-mix inclusion, PSB+ OLG-mix. All diets contained a commercial enzyme complex with β-glucanase activities (Nutega S.L., Madrid, Spain). For the formulation of the diets, it was accepted that the inclusion of the enzyme complex increased by 2% the apparent metabolizable energy (AME) content of the wheat [28]. The experimental diets were formulated according to the recommendations of FEDNA [29] and they were isoenergetic and isoproteic.

The products added to the diets at 2 g/kg were provided by a commercial company (Novation Co., Soria, Spain). PSB contained 54% sodium butyrate protected by a physical and chemical matrix of buffer salts (21% sodium). Cooling after the reaction of butyric acid with buffer salts resulted is a protected chemical structure that avoided the release and absorption of the free acid in the upper part of the gastrointestinal tract. OLG-mix was mainly composed by 62% of olive tree leaves and 24% of grape by-products. The main bioactive compounds of OLG-mix were hydroxytyrosol (HXT), oleuropein (OLE), resveratrol (RES), and maslinic acid (MA), followed to a lesser extent by α-pinene (α-P), thymol (TYH), and carvacrol (CAR). The concentration of total phenolics and terpenes of the OLG-mix was analyzed as quality control in order to fulfill a minimum concentration of 7000 mg/kg and 70,000 mg/kg, respectively (Table 1). No coccidiostat, antibiotic, or any other growth promoter were used. From 0 to 12 days of age, the diet was provided in crumble form, and then from 13 to 28 and from 29 to 40 days of age, diets were presented in pellet form. Feed and water were provided ad libitum during all the experiment.

### 2.4. Experimental Procedures

Body weight (BW) and feed disappearance were recorded by replicate at 0, 13, 29, and 40 days of age. From these data, average daily gain (ADG), average daily feed intake (ADFI), and feed conversation ratio (FCR) were calculated for each period and cumulatively. In addition, mortality was recorded and weighted as produced. The European Broiler Index (EBI) was calculated for the global period (0–40 days) according to the equation: ADG (g/day) × viability (%)/FCR × 10.

At the end of the trial, three broilers per pen were randomly selected, individually identified, and BW recorded. Carcass weight (CW) was measured after processed in a commercial slaughterhouse by removing the feathers, blood, head, feet, abdominal fat (fat surrounding the cloaca and gizzard), and all the viscera. The carcass yield (CY), as the relationship between the BW and the CW at slaughter, was expressed as the g/kg of CW or the live BW. Breast and thigh muscle were excised and weighed with accuracy of 0.1 g (Gram precision RZ/6, L’Hospitalet de Llobregat, Barcelona, Spain) to calculate the breast and thigh yield (BY and TY, respectively) and based on BW or CY. The right part of breast muscle of one broiler per pen was used to analyze meat color and drip loss, and the left part was intended for thiobarbituric acid reactive substances (TBARs) analysis.

### 2.5. Laboratory Analysis

#### 2.5.1. Diets Composition

Feed components were analyzed using official standard methods: for moisture (method 930.15), ash (942.05), crude protein (984.13), ether extract (920.39), and crude fiber by the filtering bag technique (962.09) [30]. Furthermore, starch content was obtained following procedure described by Ratanpaul et al. [31], and the mineral composition was evaluated after mineralization with inductively coupled plasma mass spectrometry (ICP-MS) [31,32] (Table 1; Appendix A).

#### 2.5.2. Total Phenolics and Terpenes Analysis of the Plant Mixture

The concentration of total polyphenols was measured following the method proposed by Blainski et al. [33]. All chemicals were analytical-reagent grade. Briefly, plants by-products were washed with water, dried in a circulating-air oven (37 ± 2 °C), and powdered in a hammer mill (Tigre ASN-5; mean diameter 0.42 mm). After that, the powder was homogenized in presence of 7:3 acetone:water (*v*/*v*) using an Ultra-Turrax (IKA, T25 digital homogenizer), filtered and washed again with 7:3 acetone:water (*v*/*v*). Solvents were removed in a rotavapor (VWR^®^ IKA, RV8, USA), lyophilized and stored at −20 °C until analysis. Total phenolics were determined by the addition of Folin–Ciocalteu reagent in a 25 mL volumetric flask and absorbance was read at 760 nm in UV/VIS spectrophotometer (Shimadzu PC-1650, Kyoto, Japan).

For the terpenes analysis an adaptation of Guinda et al. method [34] was used. The leaves of the OLG-mix were washed and dried in an oven at 35 °C for 24 h. After that, leaves were crushed, washed with hexane using a Soxhlet (Raypa SX, Terrassa, Barcelona, Spain), and the extract obtained was filtered. In the second phase, a new extraction was made with ethyl acetate and filtered. Finally, the content of triterpenes in the sample were quantified by HPLC-UV/vis (nucleosil C18-column) (Agilent Tecnologies 1200, Agilent Technologies, Waldbronn, Germany), using a mobile phase of methanol/water (5:1, *v*/*v*) and the detector was placed at 254 nm. Gallic acid was used as standard, and results were expressed as mg/kg gallic acid equivalents (GAE).

#### 2.5.3. Plasma Oxidative Status

At the end of the trial, one chicken per pen was randomly selected to take blood samples. Blood (4 mL) was taken in heparinized tubes and centrifuged (600× *g*, 10 min) to obtain plasma for further analysis. The contents of TBARs, α-tocopherol, ferric reducing antioxidant power (FRAP), and different antioxidant enzymes such as catalase (CAT), superoxide dismutase (SOD), and glutathione peroxidase (GPx) were measured as markers of oxidative stress.

Plasma TBARs levels were obtained following the method described elsewhere [35] using a microplate reader (Powerwave XS, Biotek instruments, Winooski, VT, USA). Results were expressed as µmoles malondialdehyde (MDA)/L plasma.

The α-tocopherol was analyzed by HPLC following the method described by Nirungsan and Thongnopnua [36]. Briefly, the plasma was deproteinized using acetonitrile and isopropanol (7:3) after the addition of α-tocopheryl acetate as internal standard. The extract containing the tocopherol was injected in an HPLC (VWR-HITACHI, EliteLaChrom) provided with a diodo array detector (LaChrom Elite system L-2455, Laboquimia, Logroño, Spain) in a C18 column (Brisa-LC2 C18, Teknokroma, Sant Cugat del Vallés, Barcelona, Spain). Methanol was used as the mobile phase and the effluent was quantified at 292 nm.

For the FRAP analysis, the principle of the method was based on the reduction of the ferrictripyridyltriazine (Fe^3+-^TPTZ) complex to ferrous tripyridyltriazine (Fe^2+-^TPTZ), leading to a blue color whose intensity was proportional to the reducing capacity of the mainly non-enzymatic antioxidants in the sample [37]. For this purpose, 300 μL of freshly prepared FRAP reagent (0.7 mM TPTZ and 1.5 mM of ferric chloride hexahydrate in an acetate buffer, pH 3.6) was mixed with 10 μL of the sample. Absorbance was read at 600 nm wavelength (Shimadzu PC-1650, Kyoto, Japan). The calibration curve was obtained with known concentrations (from 0.1 to 1.0 mM) of Fe (II) solution and results were expressed in mmoles Fe (II) equivalents/L.

The measurement of CAT activity was based on the automated method described by Slaughter and O’Brien [38]. The production of H_2_O_2_ was measured with the Trinder reagent (4-aminophenazone and 3,5-dichloro-2-hydroxybenzenesulphonate) and horseradish peroxidase. The calibration curve was produced by using a series of catalase standards prepared in Tris-buffer (20 mM, pH 7.4). The levels of SOD and GPx were determined using the commercial kits Ransod and Ransel (Randox Labs, Crumlin, UK) according to manufacturer instructions. Enzymes were analyzed in an Olympus AU600 automated chemistry analyzer (Olympus Diagnostica Europe GmbH, Ennis, Ireland). The concentrations were calculated as Units (U)/mL or U/L of the corresponding enzyme.

#### 2.5.4. Drip Losses in Muscle Samples

Drip losses were estimated by the suspension method [39]. Approximately 15 g of samples were taken from the *Pectoralis major* muscle. After cutting, samples were weighed, put inside of a mesh and a plastic container that was placed under refrigerated conditions at 4 °C. Samples were weighed again at 24 h of storage. The difference between final and initial weights was used to calculate the drip losses that was expressed as a percentage of the initial weight.

#### 2.5.5. Determination of the Oxidative Stability of the Muscle by the TBARs Method

TBARs, as a lipid peroxidation index, were measured in breast muscle following the method of Salih et al. [40]. Breast samples were placed on polystyrene trays, over-wrapped with an oxygen-permeable polyvinyl chloride (PVC) and kept at 4 °C under fluorescent light (616 l×). At fixed time intervals (0, 3, and 6 days) meat samples were mixed with a perchloric acid solution (3.83%) and 0.5 mL of BHT (4.2% in ethanol) using a mixer mill (MM400, Retsch Technology, Haan, Germany). Then, samples were centrifuged at 600× *g* during 10 min at 4 °C, and aliquots were added to TBA (0.02 M) in a 1:1 ratio and heated during 15 min in boiling water. Absorbance was measured spectrophotometrically at 532 nm (ScanGo, ThermoFisher Scientific, Alcobendas, Spain). The TBARs values were expressed as mg of malondialdehyde (MDA)/kg of muscle, comparing the absorbance with a standard curve made with 1.1.3.3-tetraethoxipropane in methanol.

#### 2.5.6. Instrumental Color Analysis

Breast color was evaluated by means of a chromameter (CR-300 Chroma Meter, Konica Minolta, Osaka, Japan) previously calibrated against a white tile. The average of three random readings was used to measure lightness (L*), redness (a*), and yellowness (b*) according to CIELAB system [41]. Conditions for measurement were D65 illuminant, observer angle 10, and 0.8 mm aperture. In addition, the chroma and hue angle were calculated following the equations: chroma = (a*^2^ + b*^2^)^0.5^ and hue angle = arctangent (b*/a*).

### 2.6. Statistical Analysis

Data of growth performance, blood oxidative status, and meat quality were analyzed as a completely randomized design using the general linear model (GLM) procedure of SAS v9.2 (SAS Institute Inc, Cary, NC, USA), that included the fixed effects of PSB and OLG-mix with their interaction in a full factorial model. The pen (22 broilers per pen) represented the experimental unit for all measurements assuming a statistical power > 0.80. When significant differences among treatments were observed, means were separated using the Tukey test. Differences were considered significant at *p* < 0.05, and values between *p* > 0.05 and *p* < 0.10 were considered as a trend. Pearson correlation analyses were carried out between performances parameters and different plasma and muscle measurements using Statgraphics-19. The relationship between plasma vitamin E and meat MDA, as well as the relationship between plasma SOD and muscle MDA according to dietary treatment were quantified by regression equations (Statgraphics-19). Differences between slopes in regression equations were analyzed by Student’s *t*-test.

## 3. Results

### 3.1. Performance Parameters

Animals supplemented with PSB in the diets had greater ADG and lesser FCR at the starter phase (0–12 days) (*p* < 0.01 and *p* < 0.001, respectively) than those un-supplemented (Table 2). Moreover, FCR tended to be lesser from 13 to 28 days (*p* = 0.0507). However, these effects disappeared with the age. As a result, ADG and FCR presented an opposite effect from 29 to 40 days of ages (*p* < 0.05). No differences were observed between treatments in ADFI throughout the experimental period.

Additionally, birds receiving OLG-mix supplemented diets showed a tendency to have lesser ADG from 0 to 12 days of age (*p* = 0.0696) than the non-supplemented, but no differences were observed during the other growing periods. However, the combination of PSB + OLG-mix produced similar ADG than PSB group during the first phase of growing. No interaction effects between treatments were observed as a result of the combination of PSB and OLG-mix on these parameters.

Dietary treatment did not modify the percentage of mortality throughout the experimental period or EBI value (0–40 days) (Table 3).

### 3.2. Carcass Parameters

No differences were observed in slaughter performances according to dietary treatment, except for thigh yield (expressed as g/kg of BW and g/kg of CY), which was lesser in animals fed with the PSB diets (*p* = 0.0413 and *p* = 0.0364, respectively) (Table 4) than in those without PSB supplementation. Neither the combination of PSB + OLG-mix produced any significant change on carcass parameters.

### 3.3. Plasma Oxidative Status

The oxidative status of blood samples is presented in Table 5. Plasma from birds supplemented with 2 g/kg PSB had greater concentrations of SOD in plasma (*p* = 0.0434) than non-supplemented groups. In addition, animals receiving OLG-mix supplementation (2 g/kg) presented greater plasma concentration of SOD (*p* = 0.0471) than those non-supplemented groups.

The combination of 2 g PSB/kg + 2 g OLG-mix/kg did not produce additive effects on SOD concentration, and plasma from these birds presented similar SOD levels of this enzyme than those from PSB or OLG-mix groups.

The other blood parameters (MDA, FRAP, CAT, GPx, and vitamin E) were not statistically affected by dietary treatments.

No interaction effects were found on plasma parameters between the experimental groups by the administration of PSB and OLG-mix.

### 3.4. TBARs Production in Meat, Drip Losses and Changes in Color Parameters

The concentration of TBARs (thiobarbituric acid reactive substances) in breast increased with the time of storage under refrigeration (Table 6). Moreover, the breast from PSB-supplemented birds had lesser content of MDA (malondialdehyde) throughout the storage time (*p* < 0.05 at 0, 3, and 6 days of refrigerated storage) than groups without PSB supplementation. Additionally, the PSB group reached the lowest MDA values in meat at days 3 and 6 of storage when compared to the other groups. However, no significant differences were observed between the groups supplemented with any of the additives.

OLG-mix supplementation did not statistically modify the MDA concentration of muscle until day 6 of refrigerated storage. However, after that storage time, meat from birds supplemented with OLG-mix resulted in a tendency to have lesser TBARs production (*p* = 0.0567) compared to groups without OLG-mix supplementation.

An interaction effect was observed at day 6 of refrigerated storage (*p* = 0.0188). The combination of PSB+ OLG-mix decreased the MDA concentration of muscle when compared to control group. However, poultry meat from groups supplemented with PSB + OLG-mix resulted in lesser TBARs numbers than the individual supplementation of OLG-mix.

The color parameters and drip losses of meat are presented in Table 7. Breast from PSB-supplemented birds tended to have greater redness (a*) and hue angle (tone) (*p* = 0.0730 and *p* = 0.0912, respectively) than meat from groups non-supplemented with PSB (Table 7). Conversely, OLG-mix group presented greater yellowness (b*) and chroma (intensity of color) in the breast (*p* = 0.0332 and *p* = 0.0318, respectively). However, the combination of PSB + OLG-mix did not produce changes in color parameters and breast from birds supplemented with PSB + OLG-mix presented similar yellowness, and chroma than those from birds receiving a single administration of PSB or OLG-mix.

No interactions between dietary treatments were observed on color measurements in the present research.

In addition, supplementation with PSB resulted in greater drip losses in meat (*p* < 0.05); whereas breast from animals fed with OLG-mix in diets tended to have the lowest drip loss values (*p* = 0.0668). No interaction between treatments was observed in any case.

### 3.5. Relationship between Plasma Antioxidant Status and Performances or Breast TBARs

**Figure 1:**
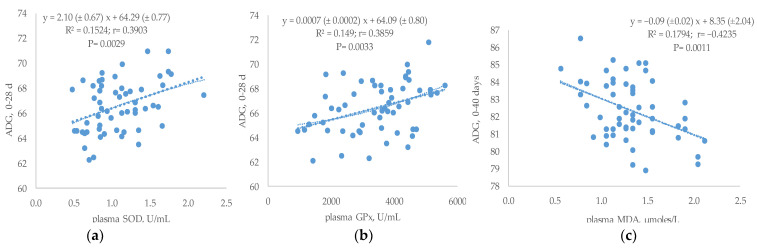
Relationship between ADG (g) and plasma antioxidant status measured as SOD (U/mL) (**a**), GPx (U/mL) (**b**) or MDA concentrations (µmoles/L) (**c**).

High correlation and linear response were found between different blood antioxidant parameters and ADG at 0–28 days or 0–40 days (Figure 1a–c). Thus, the ADG during the initial growing period (0–28 days) was directly correlated with plasma SOD (r = 0.39; *p* = 0.0029) and GPx (r = 0.39; *p* = 0.0033) concentrations. Moreover, the ADG for whole growing period (0–40 days) was negatively correlated with plasma MDA (r = −0.42; *p* = 0.0011).

**Figure 2:**
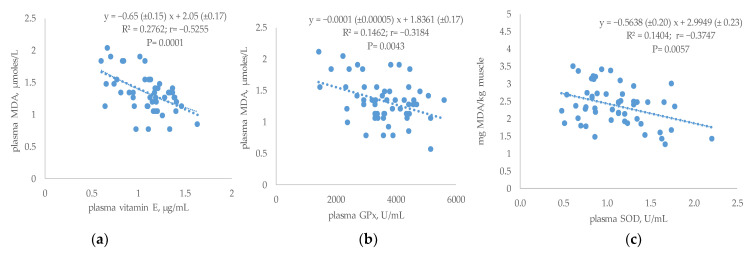
Relationship between plasma MDA (µmoles/L) and plasma vitamin E (µg/mL) (**a**) or GPx (U/mL) (**b**); and between muscle MDA (mg/kg) and plasma SOD (**c**).

In addition, inverse relationships were observed between plasma MDA and other blood compounds as indicators of antioxidant status (Figure 2a,b). As a result, plasma MDA concentration was negatively correlated with plasma vitamin E (r = −0.53; *p* = 0.0001) (Figure 2a) and with GPx (r = −0.32; *p* = 0.0043) (Figure 2b) following a linear adjustment.

The plasma oxidative status was also correlated with breast lipid oxidation (Figure 2c). The enzyme that presented the highest correlation value was SOD, that was linear and negatively correlated with the MDA concentration of meat (r = −0.37; *p* = 0.0057).

The relationships between plasma MDA and vitamin E and between muscle MDA and plasma SOD were affected to a different extent by the dietary treatment (Table 8). As a result, plasma MDA suffered a lesser increase when plasma vitamin E decreased in groups supplemented with 2 g/kg OLG-mix compared to those that did not receive OLG-mix supplementation. Whereas the drop slopes quantified by the regression equation between plasma MDA and vitamin E were similar in PSB supplemented groups than in those non PSB-supplemented.

Moreover, changes in muscle MDA concentration according to SOD concentration were also affected with different intensity by the dietary treatment. Thus, the negative slopes quantified between muscle MDA and plasma SOD differed in PSB-supplemented groups compared to those un-supplemented (Table 8). As a result, the PSB supplemented groups showed lesser response to muscle MDA concentration with the variation in SOD than the unsupplemented groups. Similarly, birds supplemented with OLG-mix had lesser negative slopes than those non-OLG-mix supplemented and then changes in muscle MDA concentration would be affected in lesser extend with the variations in plasma SOD.

## 4. Discussion

There is no previous research comparing the use of dietary PSB supplementation with the administration of a mixture of phytobiotic compounds, mainly derived from olive leaves or grape by-products. For the mixture of phytobiotics, the previous effects observed in the literature based on their bioactive compounds and the antioxidant action [19,20], were taken into account. Furthermore, considering the potent and positive effect of supplementation with polyphenolic extracts derived from olive leaf or grape by-products on the oxidative status and quality of meat [19,20,24,35], it was considered for the present investigation one of the highest doses of PSB described in the literature (2 g/kg), even though lower doses could have significant effects on different parameters [15,16].

### 4.1. Effect of PSB and OLG-Mix Supplementation in Diets on Performance Parameters

The positive effects on the growth performance produced by the inclusion of PSB in diets were mainly observed in the starter phase (0 to 12 days of ages). Afterwards, these positive effects were disappearing with the age (29 to 40 days). Many studies on dietary butyrate supplementation have reported positive effects on digestive health and development, since it affects nutrient digestibility, digestive enzyme secretions, gut microbiota, and epithelial integrity, and serves as primary energy provider for colonocytes [5,9,42]. However, as observed in the present study, some authors have described non-effect to dietary supplements under favorable state of health and environment. In that way, Leeson et al. [43] reported a low growth response to coated BA (as a mixture of mono-, di-, and triglycerides) under good health conditions. Hu and Guo [44] using SB from 0.5 g/kg to 2 g/kg found a linear weight gain increase during the first growing phase (0–21 days), without any effect in whole period. Similar effects were observed by Gao et al. [16] in supplemented birds (1 g/kg of 30% microencapsulate SB). In contrast, Lan et al. [13] using 0, 0.3, 0.6, and 1.2 g/kg of PSB (containing 54% SB), found an improvement of ADG in different periods as well as the overall period. Additionally, Ogwuegbu et al. [45] using PSB at 2 g/kg found positive effects on body weight and feed conversion, but these authors used SB made up mono and diglycerides (80% butyrate). Discrepancies between studies could be in part explained by the doses used, associated with the administration form of butyrate and its availability, or to the optimal conditions that the animal reach with age, when it grows at a slower rate. Thus, the growth of the main components of the gastrointestinal tract and the maturation of the intestinal mucosa occurs in chicks during the first 10 days of life, while adequate secretion of digestive enzymes takes about 21 days to reach optimal levels [46]. The growth-promoting action of butyrate would therefore be more effective when the chick’s digestive system is less developed. In addition, PSB has been described as a cellular mediator regulating multiple functions of gut cells, and thus produces an increase in the villus height and a larger surface area that increases the absorption capacity [5,13].

Otherwise, the action of the OLG-mix supplement at 2 g/kg did not present any effects on performance parameters in the present study, except for the tendency to a lower ADG observed in the starter phase (0–12 days of age). The non-significant effects of OLG-mix on feed intake would suggest that phytobiotic extract did not adversely affect the palatability of the diet. Sierzant et al. [47] observed that the use of 2.5 or 5 g/kg of olive leaves, rosemary, or pine bark did not modify the ADG or FCR of birds from 1–32 days of age. Additionally, Xie et al. [48] did not report any effect on ADG and ADFI after the supplementation with 1–3 g/kg of olive leaf extract. However, the addition of 4 or 5 g/kg dramatically reduced these performance parameters in the early period (1–21 days), because of the bitter taste of oleuropein that would affect palatability of the diet [48]. Other studies [49] that evaluated the use of olive leaf or grape seed, predominant ingredients in the OLG-mix, at lower doses (0.1–0.2 g/kg) reported that the use of olive leaves produced increases in ADG and improved FCR during the first 6 weeks of life when compared to the use of grape seed-supplemented diets (0.1–0.2 g/kg) or the control group. Some authors have reported negative impacts of polyphenols on performances when added in relatively high concentrations in poultry diets attributed to a decrease in fat and protein digestion by binding salts, inactivating digestive enzymes, or both [50].

On the other hand, the combination of both supplements in the present study did not modify performance parameters throughout the growth period. To our knowledge, there is no study in the literature in which PSB is combined with a plant/fruit-based supplement in broiler feeding. In a study in which PSB was combined with a unique leaf meal from a plant (rosemary), authors reported that the unique administration (butyrate or rosemary) and lower doses of rosemary leaf (2.5 g/kg) had more positive effects on performances than in combination [45].

### 4.2. Effect of PSB and OLG-Mix Supplementation in Diets on Carcass Parameters

In the present assay, no differences between the inclusion or non-inclusion of PSB in diets were found on carcass yield except for the relative thigh yield which decreased with PSB supplementation. In this context, Yang et al. [51], using lower supplementation dose of microencapsulated butyrate (1 g/kg, containing 30% pure SB) than the used in the present research, did not find significant effect on the yields of carcass and breast from chickens. Additionally, Lan et al. [15] did not find any effect on carcass traits from birds given 0.3–1.2 g/kg PSB. Conversely, Mátis et al. [52], using very different SB forms than the used in the present research, observed an increment of carcass weight and elevated breast meat yield by the use of distinct protected forms of butyrate (film-coated with 90% SB content at 1 g/kg diet; vegetable fat-embedded with 40% SB content at 1.5 g/kg diet or 30% SB content at 2 g/kg diet) when compared to controls. The relative changes in thigh yield observed in the present study could be explained by the fact that thigh accumulates higher amount of fat than other muscles; however, butyrate supplementation has been reported to enhance oxidation of lipids and decreased body fat deposition [53]. Therefore, changes in relative thigh weight would be more marked than in other muscles.

The supplementation with phytobiotics additives did not alter carcass parameters which is supported by results of other authors when using these ingredients at low doses [54,55].

### 4.3. Effect of Dietary PSB and OLG-Mix Supplementation on Oxidative Status of Broilers

The control of oxidative stress is a relevant aspect to achieve adequate growth and a good state of health [56]. Under physiological conditions, all cells produce reactive oxygen species (ROS) that can cause cellular damage, if they are not captured in time by the body’s antioxidant systems [57]. Therefore, an imbalance in the antioxidant and oxidant capacity of the organism can lead to the appearance of diseases and physiological alterations. One of the cellular antioxidative systems consists of enzymes (CAT, SOD, GPx) with capacity for quenching ROS [56]. In the present study, the PSB or OLG-mix supplementation at 2 g/kg produced protective effects against oxidative stress since it was observed higher SOD activity in plasma of birds supplemented with one or the other compound. Lan et al. [15] also reported a major activity of SOD and GPx in serum of birds supplemented with PSB (54% of SB protected by a physical and chemical matrix of buffer salts), at different levels (0.3, 0.6, and 1.200 g/kg). In the same way, the use of 0.4 g microencapsulated SB/kg diet decreased the MDA concentration and increased the activity of CAT in serum from broilers induced to stress [14]. The protective mechanisms of butyrate on the control of oxidative stress are not entirely clear. It has been found that SB protects against oxidative stress in HepG2 cells through modulating energy metabolism, mitochondrial function, and nuclear factor erythroid 2-related factor 2 (Nrf2) [58]. In a recent study carried out in vivo, Sun et al. [59] reported that the abolition of the MDA concentration and major activity of GSH and SOD in rats given SB supplementation was also due to the induction of the transcription of the Nrf2. The Nrf2 is an important molecular switch that orchestrates the gene expression of antioxidant and phase II enzymes [60] to reduce oxidative stress.

To our knowledge, no information is available on the oxidative stress in broilers by the supplementation with similar doses of PSB to those used in the present study. However, it is interesting to highlight that the administration of 2 g/kg PSB produced similar effects to control oxidative stress than the administration of the same dose of OLG-mix. The use of several plants or fruits-based by-products and their extracts have been widely documented as a feeding strategy to reduce oxidative stress [50,61]. Hence, exogenous antioxidants such as polyphenols, enhance free radicals removing mechanism by a strong scavenging activity and antioxidant enzymes activation [50,61]. In addition, several studies indicate that the use of olive or grape by-products or their active compounds (OLE, HXT, RES) also activate the Nrf2 pathway, for example SB [60,62,63,64,65,66]. The antioxidant activity of these compounds has been observed even at low doses. Therefore, the supplementation with 0.2 g/kg of olive leaves improved the oxidative status of birds by the increase in SOD and GPx [67]. Additionally, Gerasopoulus et al. [68], using by-products from olive oil mill wastewater, reported an enhance in broilers’ redox status associated with an increase in CAT and glutathione enzymes. Moreover, the use of other olive by-products resulted in higher activity of SOD and CAT in the liver of broilers fed with a diet containing extra-virgin olive oil, which was related to the activation of these enzymes by the HXT [69]. The use of grape seed extract has also been effective to restore the balance in the antioxidant/oxidant system after an infection [70] with an increase in plasma SOD. Considering the antioxidant effects of the phytobiotics and PSB administered individually in the present study, it would be expected that a combination of both compounds might produce more substantial effects; however, no significant changes on oxidative stress were observed because of the combined administration of PSB + OLG-mix. It has been reported that polyphenols in some environments may exert pro-oxidant effects or interact in the gut mainly when are administered with other antioxidants [50,61]. Thus, in the present research, the effects of one of the supplements could be counteracted by the presence of the other, which would explain the lack of additive effects on the oxidative status of the birds.

It is interesting to remark that the improvement in oxidative status observed in the present study was directly related to the ADG at the initial or total growth period. Thus, SOD and GPx concentrations were linearly and positively correlated with the ADG from 0–28 days, whereas plasma TBARS concentration correlated negatively and followed a significant linear fit with the ADG for the whole growing period (0–40 days). It is widely documented that an alteration in the control of the oxidant/antioxidant balance could negatively affect the immune response [71] and nutrient expenditure [72] with the consequent negative effects on productive performances. However, there is a lack of information in the literature on the prediction of ADG in function of the oxidative status of blood. Hung et al. 2017 [73] in a meta-analysis study found that feeding peroxidized lipids reduced growth performances of broilers and suggested the need to develop a more accurate measurement to predict the negative impacts of an increase in oxidation rate. The present study would therefore be a first approximation to the possibilities of quantifying growth range from the evaluation of the blood oxidative status.

Furthermore, although only changes in plasma SOD concentrations were observed, significant correlations were found between plasma TBARs and GPx or vitamin E. Moreover, changes in linear response between TBARs and vitamin E were also different between treatments. Thus, the increase in plasma TBARs with lower concentrations of plasma vitamin E in OLG-mix supplemented groups was of lesser magnitude than those that did not receive OLG-mix supplementation. These results would indicate the presence of other antioxidant compounds that might maintain the oxidative status and the plasma vitamin E levels in OLG-mix groups. However, drop slope was similar between PSB-supplemented and PSB un-supplemented groups which would indicate the absence of additional antioxidants to better preserve the plasma vitamin E. Other authors have also reported the increased concentration of vitamin E associated with polyphenol supplementation [61] and the positive effects that this entails, since vitamin E is a very powerful antioxidant in the body [35,61].

### 4.4. Effect of Dietary PSB and OLG-Mix Supplementation on Meat Lipid Stability

Getting to prolong the shelf life of meat is a fundamental aspect at the industry level and for the consumer. This is especially relevant in chicken meat, which due to its high PUFAs content is very prone to oxidation [17]. In the present study, 2 g/kg of PSB supplementation delayed the lipid peroxidation of the breast with a lower content of MDA along all the days of valuation (0, 3, and 6 days), extending the shelf life of the meat and preserving their quality. Other authors using lower doses of PSB [15,16] described a reduction of MDA content in breast muscle, indicating that SB supplementation decreased lipid peroxidation. In a similar way, Zhang et al. [14] using, 0.4 g/kg of microencapsulated SB, observed a decrease of MDA levels and an increase of CAT activity in breast muscle of broilers subjected to stress by experimental corticosterone injections [14]. As far as we know there is not further information in the literature. It is interesting to remark in the present study that the effect of PSB to preserve oxidation of muscle was more effective and longer over time than the use of OLG-mix. This is a relevant result since the use of plant derivatives to preserve the shelf life of meat is well documented [61,74]. However, there is controversy in the doses of administration since it has been observed that very high doses could hinder the bioavailability of these compounds and therefore their effects on meat quality [50,61]. In the present study, the supplementation with OLG-mix produced a trend towards a reduction in the MDA levels of breast muscle after sixth valuation day. Other authors [24,48] found OLE (the main component of OLG-mix used in the present study) at doses between 0.1–0.3 g/kg as an effective supplement in diets to control lipid oxidation of meat. Additionally, grape by-products have been reported as natural sources of polyphenols and vitamin E [75] and may improve oxidative stability of chicken meat [55,75,76,77]. The effects of polyphenols on the oxidative stability of meat have been observed in many studies after more than 6 days of refrigerated storage when meat begins to lose its shelf life for consumption [22,55,75,77], as has been observed in the present study. Then, a delay in meat oxidation would provide indirect evidence that these antioxidants compounds could be absorbed.

In relation to the effectiveness of the combination of PSB and phytochemical compounds, there is a lack of information in the literature. According to the results of the present study, the combination of OLG-mix with PSB seemed to be more effective to control lipid oxidation of meat than the unique use of OLG-mix, mainly after 6 days of storage (interaction effect). The mechanism of PSB to preserve the oxidative stability of meat is unknown; however, it could be related not only to presence of antioxidant enzymes [15] but also to changes in lipid metabolism and meat composition. Thus, Heimann et al. [78] reported that butyric acid may inhibit lipolysis and *novo* lipogenesis as well as decreased glucose uptake in adipocytes, which means less circulating free fatty acids. An excess in free fatty acid production has been associated with increased lipid oxidation and meat quality deterioration [24,79].

In addition, a significant linear relationship was observed in the present study between plasma SOD and muscle MDA. This relationship changed according to dietary treatments and was quantified by regression equations. In both cases, the supplemented groups presented a significantly different drop slope compared to their non-supplemented. Therefore, a decrease in blood SOD would produce a more marked increase in muscle TBARs than when birds received a supplement. These results would indicate that in both cases, the stability of the meat to oxidation might be explained to a greater extent by the oxidative status in vivo as stated by other authors [15,24,61]. However, changes between supplemented and non-supplemented of the corresponding groups (differences in the slopes) were greater for OLG-mix than for PSB, what would confirm that in PSB other factors and not only in vivo oxidative status would be involved. Results could be an initial approximation to predict TBARS levels in meat from plasma SOD and thus be able to establish adequate control measures when the animal has not yet been slaughtered.

### 4.5. Effect of PSB and OLG-Mix Supplementation in Diets on Breast Meat Color and Drip Losses

The color of the meat is one of the main quality attributes and directly influences in the consumer choices. The raw chicken breast color is slightly pinkish but can also appear bluish white to yellow due to several factors [80]. It has been reported that the color of the meat could be affected by the concentration, chemical, and physical state of the myoglobin, as well as the structure of the meat surface [80,81]. In the present study, dietary PSB supplementation with 2 g/kg slightly modified the color of the breast presenting a tendency to greater redness (a* value). Gao et al. [82] found that the supplementation with 5 g/kg SB produced a deeper red color in the butyrate group than in the control group. After fiber analysis these results were attributed to a higher amount of the type I fibers and myoglobin content in skeletal muscle [82]. Additionally, greater redness values by butyrate supplementation may be related to an increase in the oxidized slow fiber to fast fiber ratio [83].

The OLG-mix supplementation also changed breast color. The bioactive substances contained in plant-based products could stabilize color by inhibition in lipid oxidation. Thus, OLG-mix supplementation increased yellowness (b* value) and color intensity (Chroma). These results are supported by the findings of other authors that used different phytobiotics compounds in animal feeding [76,84]. Color changes might be related to the type of carotenoids presents in plant/fruits-based by-products being xanthophylls the most abundant [84]. The greater intensity in yellowness in chicken meat would be more appreciated by consumers since the color would be closer to that of the meat of birds raised under freedom conditions [85].

The luminosity of meat (L*) which has been related to water losses [79] were not affected by any dietary treatment. Unexpectedly, water-holding capacity decreased in meat from PSB-supplemented birds. These results are contrary to what was observed by other authors that administered PSB at lower doses than the used in the present study [15,16]. These discrepancies could be related to differences in fat metabolism by a higher dose of PSB supplementation [53]. The increased lipolytic activity and lower *novo* synthesis pathway reported in butyrate-supplemented birds [53] could affected glucose utilization, glycolysis and lactic acid formation in muscle, and consequently, different extent of pH falls and drip loss [79,86]. Therefore, during lipogenesis process, glucose is converted to fatty acids [87]; however, inhibition in lipogenesis might have resulted in higher muscle glycolic potential.

Concerning the use of plant/fruit-based supplements, it was observed that dietary OLG-mix supplementation seemed to decrease drip losses (although differences did not reach significant levels), which is related to enhance in the quality of meat. The use of 0.1 or 0.2 g/kg of olive leaves or grape seed extracts have been found to be effective to reduce drip losses of chicken at 3 days of storage [77]. Balzan et al. [88] also observed a decrease in drip loss values with time in birds fed diets with olive vegetation water at the lowest dose of polyphenols compared to control. Moreover, Zhang et al. [89] found a reduction of drip losses 24 h postmortem in broilers supplemented with resveratrol. The positive effects of different lipophilic or hydrophilic antioxidant compounds on water-holding capacity of meat have been attributed to the greater proteolysis in muscle due to the protective effect on some protease enzymes [90]. However, the combination of both dietary supplements did not alter drip losses of meat in the present study, probably due to the different mode of action on metabolism and the contrary effects of each other.

## 5. Conclusions

To conclude, the supplementation of PSB at 2 g/kg was more effective than OLG-mix supplementation to improve productive parameters during the initial growing period, without deleterious effects on whole growing phase. Both PSB and OLG-mix showed similar protective effects against oxidative stress in vivo, but PSB supplementation was more effective to delay the lipid oxidation of meat from the initial day of storage. However, the use of phytobiotics from olive leaf and grape derivatives improved some meat quality characteristics, such as color and drip losses, to a greater extent than PSB supplementation. The combination of both compounds did not have more marked effects that the individual administration; except to control the oxidation of meat. The oxidative status of the birds was highly correlated with weight gain and meat stability. Therefore, it would be possible to predict performances and shelf life of meat through the plasma oxidative status.

## Figures and Tables

**Table 1:** Calculated composition and analysis of olive and grape-based by-product (OLG-mix) (g/kg as fed basis, unless otherwise indicated).

Calculated Composition	OLG-Mix
Ash	139.0
Crude protein	56.0
Ether extract	92.0
Crude fiber	329.0
Digestible Lysine	1.0
**Analyzed composition**	**OLG-mix**
**(mg GAE/kg)**	
Polyphenols	7000
Terpenes	70,000

**Table 2:** Effects of dietary protected sodium butyrate (PSB) and/or olive leaf and grape-based by-product (OLG-mix) and their interaction on average daily gain (ADG, g), average daily feed intake (ADFI, g), and feed conversation ratio (FCR) in starter, grower, and finisher phases in broilers.

PSB (g/kg)	OLG-Mix (g/kg)	0–12 Days				13–28 Days				29–40 Days				0–40 Days		
		ADG	ADFI	FCR		ADG	ADFI	FCR		ADG	ADFI	FCR		ADG	ADFI	FCR
0	0	33.1 ^b^	39.2	1.19 ^a^		91.1	127	1.40		121 ^a^	211	1.75 ^b^		82.1	125	1.52
2	0	33.6 ^a^	38.8	1.16 ^b^		92.5	127	1.38		117 ^b^	209	1.79 ^a^		81.8	124	1.52
0	2	32.5 ^c^	38.6	1.19 ^a^		90.5	127	1.40		121 ^a^	212	1.75 ^b^		82.1	125	1.53
2	2	33.3 ^ab^	38.5	1.16 ^b^		91.9	127	1.38		117 ^b^	210	1.80 ^a^		81.3	124	1.53
Main effects																
PSB																
	0	32.8	38.9	1.19		90.8	127	1.40		121	211	1.75		82.1	125	1.53
	2	33.4	38.7	1.16		92.2	127	1.38		117	210	1.79		81.5	124	1.52
OLG-mix																
	0	33.3	39.0	1.17		91.8	127	1.39		119	210	1.77		82.0	125	1.52
	2	32.9	38.6	1.17		91.2	127	1.39		119	211	1.77		81.7	125	1.53
SD ^1^		0.936	1.109	0.023		3.627	3.075	0.040		6.454	6.378	0.074		1.479	2.355	0.021
*p*-value ^2^																
General		0.0131	0.4172	< 0.0001		0.4605	0.9474	0.2641		0.1583	0.7201	0.1495		0.3700	0.369	0.858
PSB		0.0068	0.4712	< 0.0001		0.1376	0.9918	0.0507		0.0253	0.3038	0.0253		0.122	0.086	0.726
OLG-mix		0.0696	0.1472	0.5616		0.5563	0.5526	0.7688		0.8669	0.6128	0.8116		0.533	0.957	0.448
PSBxOLG-mix		0.5085	0.6575	0.7432		0.9912	0.9408	0.9314		0.7804	0.9401	0.6589		0.561	0.703	0.819

^1^ SD: standard deviation (*n* = 30 replicates for each main effects, *n* = 15 replicates for the interaction). ^2^ *p*: differences were statistically significant when *p* < 0.05; ^a, b, c^. Letter with different superscript were statistically significant.

**Table 3:** Effect of protected sodium butyrate (PSB) olive leaf and grape-based by-product (OLG-mix), and their interaction on mortality (%) and European Broiler Index (EBI).

PSB (g/kg)	OLG-Mix (g/kg)	Mortality (%)						EBI
		0–12 Days	13–28 Days	29–40 Days	0–40 Days			0–40 Days
0	0	0.91	1.55	0.65	3.03			522.12
2	0	1.52	1.07	1.07	3.85			513.58
0	2	0.61	1.23	0.65	2.42			521.09
2	2	0.97	1.24	0.85	3.31			512.21
Main effects								
PSB								
	0	0.76	1.39	0.65	2.73			521.60
	2	1.24	1.15	0.96	3.58			512.90
OLG-mix								
	0	1.21	1.31	0.86	3.44			517.85
	2	0.79	1.23	0.75	2.87			516.65
SD ^1^		2.278	2.136	1.833	4.011			26.283
*p*-value ^2^								
General		0.744	0.9447	0.9242	0.8233			0.3903
PSB		0.4155	0.6923	0.5489	0.4443			0.2329
OLG-mix		0.4801	0.9022	0.8258	0.6048			0.8684
PSBxOLG-mix		0.8418	0.676	0.8306	0.9763			0.9812

^1^ SD: standard deviation (*n* = 30 replicates for each main effects, *n* = 15 replicates for the interaction). ^2^ *p*: differences were statistically significant when *p* < 0.05.

**Table 4:** Effect of protected sodium butyrate (PSB) olive leaf and grape-based by-product (OLG-mix), and their interaction on carcass yield (CY), breast yield (BY) and thigh yield (TY), expressed by g/kg of body weight (BW) and g/kg of CY at slaughter (40 days of age).

PSB (g/kg)	OLG-Mix (g/kg)	CY		BY			TY			
				BW	CY		BW	CY		
0	0	799		209	262		202 ^a^	253 ^a^		
2	0	798		206	258		194 ^b^	244 ^b^		
0	2	799		206	258		198 ^ab^	248 ^ab^		
2	2	799		205	257		194 ^b^	243 ^b^		
Main effects										
PSB										
	0	799		208	260		200	250		
	2	798		206	258		194	243		
OLG-mix										
	0	799		208	260		198	248		
	2	799		206	257		196	245		
SD ^1^		0.998		1.087	1.372		1.105	1.303		
*p*-value ^2^										
General		0.9837		0.7215	0.7526		0.1470	0.129		
PSB		0.7616		0.4443	0.5053		0.0413	0.0364		
OLG-mix		0.8889		0.4780	0.4619		0.4005	0.3598		
PSBxOLG-mix		0.8300		0.6316	0.6520		0.4834	0.5006		

^1^ SD: standard deviation (*n* = 30 replicates for each main effects, *n* = 15 replicates for the interaction). ^2^ *p*: differences were statistically significant when *p* < 0.05; ^a, b^. Letter with different superscript were statistically significant.

**Table 5:** Effects of protected sodium butyrate (PSB) and olive leaf and grape-based by-product (OLG-mix), and their interaction on different blood antioxidant parameters (40 days of age).

PSB (g/kg)	OLG-mix (g/kg)	MDA (µmol/L) ^1^	FRAP (mmol/L) ^2^	SOD (U/mL) ^3^	CAT (U/mL) ^4^	GPx (U/L) ^5^	Vit E (µg/mL) ^6^
0	0	1.33	0.76	0.83 ^b^	0.05	3.58	1.04
2	0	1.35	0.72	1.14 ^a^	0.10	3.76	1.10
0	2	1.34	0.80	1.13 ^a^	0.04	3.07	1.12
2	2	1.32	0.73	1.21 ^a^	0.08	3.62	1.12
Main effects							
PSB							
	0	1.33	0.78	0.98	0.04	3.33	1.08
	2	1.34	0.73	1.17	0.09	3.69	1.11
OLG-mix							
	0	1.34	0.74	0.98	0.08	3.67	1.07
	2	1.33	0.77	1.17	0.06	3.35	1.12
SD ^7^		0.361	0.236	0.354	0.120	1.004	0.245
*p*-value ^8^							
General		0.9955	0.8104	0.0260	0.4837	0.3241	0.7835
PSB		0.9507	0.4107	0.0434	0.1518	0.1895	0.6493
OLG-mix		0.9328	0.6526	0.0471	0.6007	0.2442	0.4040
PSBx OLG-mix		0.8176	0.7734	0.2131	0.7818	0.5006	0.6719

^1^ MDA: malondialdehyde. ^2^ FRAP: ferric reducing antioxidant. ^3^ SOD: superoxide dismutase. ^4^ CAT: catalase. ^5^ GPx: glutation peroxidase. ^6^ Vit E: vitamin E. ^7^ SD: standard deviation (*n* = 30 replicates for each main effects, *n* = 15 replicates for the interaction). ^8^ *p*: differences were statistically significant when *p* < 0.05; ^a, b^. Letter with different superscript were statistically significant.

**Table 6:** Effects of protected sodium butyrate (PSB) and olive leaf and grape-based by-product (OLG-mix), and their interaction on the oxidative stability of meat (mg MDA/kg of sample) at 0, 3, and 6 days of refrigerated storage (4 °C).

PSB (g/kg)	OLG-Mix (g/kg)	0 Days ^1^	3 Days ^2^	6 Days ^3^
0	0	0.36 ^a^	1.00 ^a^	1.79 ^a^
2	0	0.30 ^b^	0.67 ^b^	1.09 ^b^
0	2	0.35 ^a^	0.91 ^ba^	1.25 ^b^
2	2	0.31 ^b^	0.73 ^b^	1.16 ^b^
Main effects				
PSB				
	0	0.36	0.95	1.52
	2	0.30	0.70	1.12
OLG-mix				
	0	0.33	0.83	1.44
	2	0.33	0.82	1.20
SD ^4^		0.080	0.356	0.475
*p*-value ^5^				
General		0.0950	0.0520	0.0005
PSB		0.0138	0.0082	0.0018
OLG-mix		0.9185	0.8044	**0.0519**
PSBxOLG-mix		0.6775	0.4287	**0.0188**

^1^ 0 days: MDA content in breast at day 0 of refrigerated storage. ^2^ 3 days: MDA content in breast at day 3 of refrigerated storage. ^3^ 6 days: MDA content in breast at day 6 of refrigerated storage. ^4^ SD: standard deviation (*n* = 30 replicates for each main effects, *n* = 15 replicates for the interaction). ^5^ *p*: differences were statistically significant when *p* < 0.05; ^a, b^. Letter with different superscript were statistically significant.

**Table 7:** Effects of protected sodium butyrate (PSB) and olive leaf and grape-based by-product (OLG-mix), and their interaction on the color of the breast in broilers at slaughter and the drip losses (g/kg) after 24 h of storage.

PSB (g/kg)	OLG-Mix (g/kg)	L* ^1^	a* ^2^	b* ^3^	hue_angle ^4^	Chroma ^5^	Drip Losses
0	0	54.64	−0.25	7.09 ^b^	−0.46	7.21 ^b^	56.0 ^ab^
2	0	56.94	0.24	8.03 ^ba^	0.49	8.11 ^ba^	67.2 ^a^
0	2	57.51	−0.07	8.73 ^ba^	−0.08	8.83 ^ba^	43.0 ^b^
2	2	56.04	0.68	9.35 ^a^	0.25	9.47 ^a^	56.9 ^ab^
Main effects							
PSB							
	0	56.14	−0.16	7.91	−0.27	8.02	49.5
	2	56.50	0.46	8.69	0.37	8.79	62.1
OLG-mix							
	0	55.77	−0.002	7.56	0.02	7.66	61.6
	2	56.78	0.31	9.04	0.09	9.15	50.0
SD ^6^		4.390	1.300	2.624	0.165	2.622	2.335
*p*-value ^7^							
General		0.3112	0.2413	0.1174	0.3105	0.1166	0.0792
PSB		0.7143	0.0730	0.2557	0.0912	0.2625	0.0484
OLG-mix		0.3881	0.3645	0.0332	0.8577	0.0318	0.0668
PSBxOLG-mix		0.1019	0.7096	0.8058	0.4140	0.8504	0.8299

^1^ L*: lightness. ^2^ a*: redness. ^3^ b*: yellowness. ^4^ Hue angle: tone of the color. ^5^ Chroma: intensity of the color. ^6^ SD: standard deviation (*n* = 30 replicates for each main effects, *n* = 15 replicates for the interaction). ^7^ *p*: differences were statistically significant when *p* < 0.05; ^a, b^. Letter with different superscript were statistically significant.

**Table 8:** Regression equations between plasma MDA concentration (µmoles/L) and plasma vitamin E (µg/mL) or between muscle MDA (mg/kg) and plasma SOD according to the dietary supplementation with protected sodium butyrate (PSB) or olive and grape-based by-product (OLG-mix).

Y	Treatment		Intercept			Variable X				r	R^2^	RSD ^1^	*p* (Lineal)
						Plasma Vitamin E (x)							
Plasma MDA	PSB-0		2.02	±	0.17	−0.64	^a^	±	0.15	−0.5255	0.2572	0.26	0.0001
Plasma MDA	PSB-2		2.08	±	0.26	−0.68	^a^	±	0.23	−0.5099	0.2600	0.30	0.0078
													
Plasma MDA	OLG-mix-0		2.33	±	0.22	−0.91	^a^	±	0.20	−0.6846	0.4686	0.25	0.0001
Plasma MDA	OLG-mix 2		2.05	±	0.23	−0.60	^b^	±	0.20	−0.5511	0.3037	0.21	0.0064
													
						Plasma SOD (x)							
Muscle MDA	PSB-0		3.45	±	0.32	−0.74	^a^	±	0.32	−0.4762	0.2268	0.44	0.0216
Muscle MDA	PSB-2		2.63	±	0.23	−0.48	^b^	±	0.18	−0.5102	0.2603	0.36	0.0129
													
Muscle MDA	OLG-mix 0		3.40	±	0.39	−0.89	^a^	±	0.37	−0.4542	0.2063	0.58	0.0258
Muscle MDA	OLG-mix 2		2.67	±	0.24	−0.40	^b^	±	0.20	−0.3752	0.1407	0.41	0.0500

^1^ SD: standard deviation (*n* = 30 replicates for each main effects, *n* = 15 replicates for the interaction). *p*: differences were statistically significant when *p* < 0.05; ^a, b^. Letter with different superscript were statistically significant.

## Data Availability

Data is contained within the article.

## Appendix A

**Table A1:** Ingredients and calculated composition of the experimental diets (g/kg as fed basis, unless otherwise indicated).

	Starter	Grower	Finisher
	(0–12 Days)	(13–28 Days)	(29–40 Days)
**Ingredients (g/kg)**			
Corn	250.0	150.0	100.0
Wheat	373.5	485.5	584.5
Molasses	0.0	5.0	5.0
Soybean meal, 47%	331.7	304.3	248.9
Soybean oil	10.5	26.3	36.0
Calcium carbonate	10.2	9.1	8.2
Monocalcium phosphate	9.0	6.6	4.4
Salt	3.0	2.9	2.9
DL-Methionine	3.0	2.5	2.3
L-Lysine HCl	2.7	1.9	2.0
L-Threonine	1.4	0.9	0.8
Vitamin and mineral premix ^1^	5.0	5.0	5.0
**Calculated composition** **(g/kg of feed) ^2^**			
Dry matter	883.5	886.1	888.1
Ash	53.4	49.6	44.5
AMEn (MJ/kg)	121.4	125.7	130.0
Crude protein	217.6	207.4	187.9
Ether extract	29.6	43.3	51.7
Crude fiber	29.6	43.3	51.7
Starch	38.5	389.7	418.0
Calcium	9.6	8.7	7.9
Total phosphorus	6.0	5.3	4.6
Available phosphorus	4.8	4.4	3.9
Sodium salt	1.4	1.4	1.4
Digestible amino acids			
Lys	11.9	10.6	9.4
Met	5.8	5.1	4.7
Met + Cys	8.9	8.1	7.5
Thr	8.0	7.1	6.3
Trp	2.3	2.2	2.0

^1^ Vitamin and mineral premix. Supplied per kilogram of diet: vitamin A, 7500 IU; vitamin D_3_, 2000 IU; vitamin E, 9 mg; vitamin K_3_, 1.5 mg; vitamin B_2_, 4 mg; vitamin B_6_, 1.5 mg; vitamin B_12_, 0.009 mg; niacin, 21 mg; calcium D-pantothenate, 6 mg; folic acid; 0.4 mg; biotin, 50 mcg; choline chloride, 350.10 mg; iron (II) sulphate monohydrate, 30 mg; potassium iodide, 1.76 mg; copper (II) sulphate pentahydrate, 6 mg; manganese (II) oxide, 75.04 mg; zinc oxide, 50.05 mg; sodium selenite, 0.201 mg; citric acid, 0.167 mg; butylated hydroxytoluene (BHT), 0.167 mg; propyl gallate, 0.167 mg; sepiolite, 0.375 g; β-glucanase, 152 U; β-xylanases-D, 1220 U; 6-phytase 500 FTU. ^2^ According to FEDNA (2019).
